# SlicerDMRI: Diffusion MRI and Tractography Research Software for Brain Cancer Surgery Planning and Visualization

**DOI:** 10.1200/CCI.19.00141

**Published:** 2020-03-27

**Authors:** Fan Zhang, Thomas Noh, Parikshit Juvekar, Sarah F. Frisken, Laura Rigolo, Isaiah Norton, Tina Kapur, Sonia Pujol, William Wells, Alex Yarmarkovich, Gordon Kindlmann, Demian Wassermann, Raul San Jose Estepar, Yogesh Rathi, Ron Kikinis, Hans J. Johnson, Carl-Fredrik Westin, Steve Pieper, Alexandra J. Golby, Lauren J. O’Donnell

**Affiliations:** ^1^ Brigham and Women’s Hospital and Harvard Medical School, Boston, MA; ^2^ Massachusetts Institute of Technology, Boston, MA; ^3^ Isomics, Cambridge, MA; ^4^ The University of Chicago, Chicago, IL; ^5^ Parietal, Inria Saclay-lle de France, Neurospin CEA, Université Paris-Saclay, Palaiseau, France; ^6^ University of Bremen and Fraunhofer MEVIS, Bremen, Germany; ^7^ University of Iowa, Iowa City, IA

## Abstract

**PURPOSE:**

We present SlicerDMRI, an open-source software suite that enables research using diffusion magnetic resonance imaging (dMRI), the only modality that can map the white matter connections of the living human brain. SlicerDMRI enables analysis and visualization of dMRI data and is aimed at the needs of clinical research users. SlicerDMRI is built upon and deeply integrated with 3D Slicer, a National Institutes of Health–supported open-source platform for medical image informatics, image processing, and three-dimensional visualization. Integration with 3D Slicer provides many features of interest to cancer researchers, such as real-time integration with neuronavigation equipment, intraoperative imaging modalities, and multimodal data fusion. One key application of SlicerDMRI is in neurosurgery research, where brain mapping using dMRI can provide patient-specific maps of critical brain connections as well as insight into the tissue microstructure that surrounds brain tumors.

**PATIENTS AND METHODS:**

In this article, we focus on a demonstration of SlicerDMRI as an informatics tool to enable end-to-end dMRI analyses in two retrospective imaging data sets from patients with high-grade glioma. Analyses demonstrated here include conventional diffusion tensor analysis, advanced multifiber tractography, automated identification of critical fiber tracts, and integration of multimodal imagery with dMRI.

**RESULTS:**

We illustrate the ability of SlicerDMRI to perform both conventional and advanced dMRI analyses as well as to enable multimodal image analysis and visualization. We provide an overview of the clinical rationale for each analysis along with pointers to the SlicerDMRI tools used in each.

**CONCLUSION:**

SlicerDMRI provides open-source and clinician-accessible research software tools for dMRI analysis. SlicerDMRI is available for easy automated installation through the 3D Slicer Extension Manager.

## INTRODUCTION

We present an open-source software suite, SlicerDMRI, that enables research using diffusion magnetic resonance imaging (dMRI) for noninvasive, in vivo investigation of the organization of the white matter in individual neurosurgical patients. dMRI measures the random molecular motion or diffusion of water molecules, which enables estimation of the underlying cellular microstructure in tissues.^1^ Brain mapping using dMRI can provide critical preoperative information,^2,3^ including preoperative maps of critical brain connections (white matter fiber tracts) and insight into the tissue microstructure that surrounds brain tumors.

White matter fiber tracts are composed of myelinated axons and are categorized as association, projection, or commissural, depending on the brain regions they connect.^4^ The larger fiber tracts can be traced in dMRI data using a computational process called dMRI tractography.^5^ These fiber tracts can then be used for preoperative planning and intraoperative neuronavigation.^2,6,7^ The use of tractography for presurgical planning has recently gained significant clinical traction.^8-11^ Visualization of the relationship between the lesion and critical tracts can aid preservation of critical motor, language, visual, and somatosensory connections during brain tumor resection.^12-14^ This is particularly important in patients who are not good candidates for an awake craniotomy.^15^ The use of tractography pre- and intraoperatively has resulted in improvements in overall survival, extent of resection, and Karnofsky performance scores while decreasing the duration of surgery during awake craniotomies and the number of intraoperative seizures related to subcortical stimulation.^3,16^

**CONTEXT**

**Key Objective**
SlicerDMRI provides open-source and clinician-accessible software tools for diffusion magnetic resonance imaging (dMRI) research in brain cancer surgery planning and visualization.
**Knowledge Generated**
We demonstrate the use of SlicerDMRI on retrospective neurosurgical patient data. We provide the clinical rationale for each analysis along with pointers to the workflow of the SlicerDMRI tools used. We begin by demonstrating a conventional diffusion tensor imaging analysis followed by advanced multifiber and automated tractography analyses. We give two examples of how SlicerDMRI, used within the platform of 3D Slicer, enables multimodal image analysis for neurosurgical planning and guidance research.
**Relevance**
One key application of SlicerDMRI is in neurosurgery research, where brain mapping using dMRI can provide patient-specific maps of critical brain connections and insight into the tissue microstructure that surrounds brain tumors. SlicerDMRI is available for easy automated installation through the 3D Slicer Extension Manager.

dMRI also provides quantitative microstructure measures. These measures allow the assessment of changes of tissue microstructure caused by tumors and the effects of treatment.^17,18^ The most popular measures used in neurosurgical patients are derived from modeling water diffusion using tensor mathematics (diffusion tensor imaging [DTI]^19^). These measures include fractional anisotropy (FA) and mean diffusivity (MD). In neurosurgical patients, alterations in FA and MD can reflect combinations of increased water content from edema and/or tumor infiltration.^20^

Brain mapping using dMRI requires sophisticated computational processing to enable visualization and quantification, and multiple software packages exist for this purpose. In fact, several packages are US Food and Drug Administration (FDA) approved and purpose-built for neurosurgical brain mapping, neuronavigation, and radiation therapy planning. However, these dedicated packages do not make cutting-edge developments in dMRI analysis available to clinicians or are specifically designed for research needs. Rather, they are designed to provide robust results within the constraints imposed by routine clinical settings and FDA approval, where there is relatively little time to analyze the data of an individual patient and where the operators of the navigation systems are typically residents with limited training in dMRI.

SlicerDMRI is a suite of clinician-accessible software tools with a strong focus on the needs of clinical researchers. SlicerDMRI has an average of > 200 downloads per month. The main functionality of SlicerDMRI (see our previous article for a technical overview^21^) includes dMRI visualization, interactive and automated tractography, tissue microstructure modeling, and quantitative analyses. SlicerDMRI can import dMRI data, including clinical Digital Imaging and Communications in Medicine (DICOM) images, research formats of Neuroimaging Informatics Technology and Nearly Raw Raster Data, and the new DICOM tractography format compatible with multiple software platforms.^22^ The open-source codebase of SlicerDMRI can be extended as needed to fit the needs of a specific research project.

SlicerDMRI is built upon 3D Slicer, a National Institutes of Health–supported open-source platform for medical image informatics, image processing, and three-dimensional visualization.^23,24^ The deep integration with 3D Slicer provides a graphical user interface to functionality that is of interest to cancer researchers. This includes real-time integration with neuronavigation equipment, access to intraoperative imaging data, and the ability to perform multimodal data fusion, as illustrated in the advanced-use cases presented in this article. In addition, 3D Slicer provides easy access to a comprehensive set of open-source scientific computing packages, which allows fast development of custom research tools. 3D Slicer has been widely used in a number of cancer research applications, including prostate, breast, brain, and lung cancers, and as a result, provides a broad set of technologies valuable to SlicerDMRI users, such as native support for linear and nonlinear spatial transformations, hardware accelerated rendering, and DICOM interoperability. An active developer and user community uses online and face-to-face communication to coordinate bug fixes, add features, and coordinate ongoing development.^25^

In this article, we focus on a demonstration of SlicerDMRI as an informatics tool to enable end-to-end dMRI analyses, with examples of the usage on retrospective neurosurgical patient data. We provide the clinical rationale for each analysis along with pointers to the workflow of SlicerDMRI tools used. We begin by demonstrating a conventional DTI analysis followed by advanced multifiber and automated tractography analyses. Finally, we give two examples of how SlicerDMRI, used within the platform of 3D Slicer, enables multimodal image analysis for neurosurgical planning and guidance research.

## SELECTED EXAMPLE NEUROSURGICAL PATIENT DATA SETS

Retrospective imaging data sets from two neurosurgical patients were selected to illustrate two different clinical scenarios: a high-grade glioma displacing the corticospinal tracts (CSTs), with significant surrounding edema, and a high-grade glioma abutting the arcuate fasciculus (AF). Preservation of these tracts is critical for voluntary motor movements (CSTs)^26^ and language function (AF).^27^ The data were acquired at Brigham and Women’s Hospital. The use of the data was approved by the Partners Healthcare institutional review board, and informed consent was obtained from the patients before scanning. All data analyses in the current work were performed retrospectively for research purposes only.

### MRI Acquisition

All magnetic resonance data were acquired on a 3T MAGNETOM Prisma MRI scanner (Siemens Healthineers, Erlangen, Germany) with a 20-channel head coil. Anatomic imaging included volumetric T2 weighted and gadolinium-enhanced T1 weighted. dMRI was acquired with a repetition time of 3,500 ms and echo time of 58 ms with a flip angle of 90°, a voxel size of 2 × 2 × 2 mm, a b value of 1,000 seconds/mm^2^, six baselines, and a matrix of 112 × 112 with 66 axial slices. Functional MRI (fMRI) was acquired as the patient performed motor and language tasks as clinically indicated, and data were processed using FDA-approved software (BrainEx; NordicNeuroLab AS, Bergen, Norway). Ultrasound data were acquired with the BK5000 system (BK Medical North America, Peabody, MA) with a slice thickness of 0.5 mm coregistered to the T1-weighted anatomic volume using the Curve navigation system (Brainlab AG, Munich, Germany) and extracted using OpenIGTLink^28^ and 3D Slicer.

### Patient Data Set 1

A 69-year-old, right-hand-dominant man with a recurrent left-sided frontoparietal glioma initially presented with a diffuse large fronto-insular infiltrative pattern lesion. A biopsy sample was found to have a WHO grade 2 astrocytoma IDH1 wild type, which was treated with chemoradiation. Follow-up imaging at 3 years revealed progression in the left-side perirolandic area, which did not respond to salvage systemic therapy. Radiographic progression and clinical deterioration (motor weakness) led to the decision to pursue surgical resection for debulking and tissue diagnosis. Image-guided left frontal craniotomy was performed with intraoperative motor- and somatosensory-evoked potentials and cortical and subcortical mapping. Gross total resection of enhancing tumor (now grade 4) was accomplished with stable motor function immediately postoperatively and improved motor function relative to baseline at 1-week follow-up.

### Patient Data Set 2

A 49-year-old, right-hand-dominant man who presented with secondarily generalized seizures and word-finding difficulties was found to have a left-sided temporoparietal tumor immediately adjacent to posterior language areas by fMRI. He underwent image-guided craniotomy and resection of tumor with gross total resection of the lesion. Pathology demonstrated high-grade glioma, WHO grade 4.

## TRADITIONAL DTI ANALYSIS FOR NEUROSURGICAL PLANNING

DTI, which models water diffusion using a tensor model, is the most widely used method for analyzing dMRI data.^1^ DTI enables scalar measurements such as FA and MD^1,19^ and single-fiber tractography that accounts for one fiber tract per voxel.^5^ In this section, we give two clinically relevant example applications using DTI analysis in SlicerDMRI for neurosurgical planning research.

### DTI Analysis for Visualization and Quantification of Tissue Microstructure

**FIG 1.**
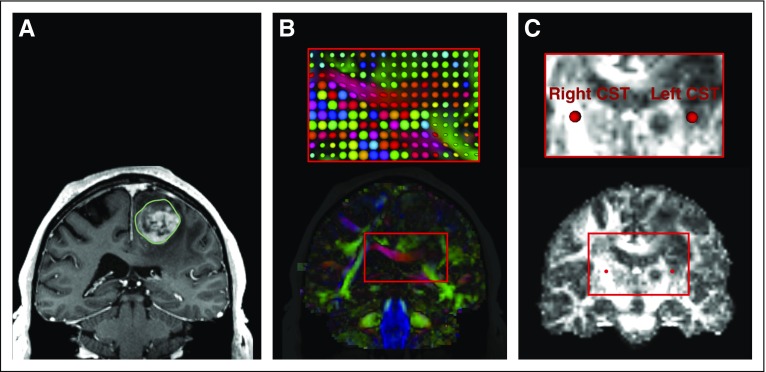
Diffusion tensor imaging (DTI) visualization and quantification in patient data set 1. The tumor-related edema and mass effect have distorted the local anatomy, including the fractional anisotropy (FA) map. (A) T1-weighted magnetic resonance imaging (MRI) with a contrast-enhancing lesion within the left frontal gyrus with the tumor volumetrically segmented in green. (B) Visualization of DTI images using a directionally encoded color map. A zoomed-in region (rectangle) is provided with a visualization using glyphs. Note that the red glyphs in the corpus callosum in the tumor-bearing hemisphere are rounder than those in the contralateral hemisphere, which indicates increased isotropic diffusion as a result of edema. (C) FA map with two fiducials in the corticospinal tract (CST; zoomed-in region outlined by the rectangle), where the FA value from the tumor-bearing hemisphere is 0.34 and that from the healthy hemisphere is 0.84.

The analysis of scalar measurements from DTI can give insight into tissue microstructure differences between the tumor and surrounding tissues. Figure 1 illustrates this analysis in patient data set 1, and the following paragraphs describe the workflow using SlicerDMRI.

#### Diffusion tensor estimation and visualization.

SlicerDMRI provides the DiffusionTensorEstimation module to calculate DTIs from diffusion-weighted image sequences. The 3D Slicer Volumes module allows visualization of diffusion tensors with multiple options, including both two-dimensional (2D) and three-dimensional (3D) displays.

#### Diffusion-derived scalar measure computation and visualization.

SlicerDMRI supports computation of quantitative diffusion measures, such as FA and MD, on the basis of the estimated diffusion tensors using the DiffusionTensorScalarMaps module.

#### Quantification of region-specific diffusion measures.

SlicerDMRI, together with other modules in the 3D Slicer core, enables quantification of region-specific diffusion measures. This can be done using 3D fiducials to define regions of interest (ROIs) and Data Probe to view diffusion measures from an ROI. This process can also be performed with the SegmentEditor module that enables manual drawing of an ROI and the LabelStatistics module that calculates statistics for different ROIs.

### DTI Tractography Analysis for Mapping White Matter Connections

**FIG 2.**
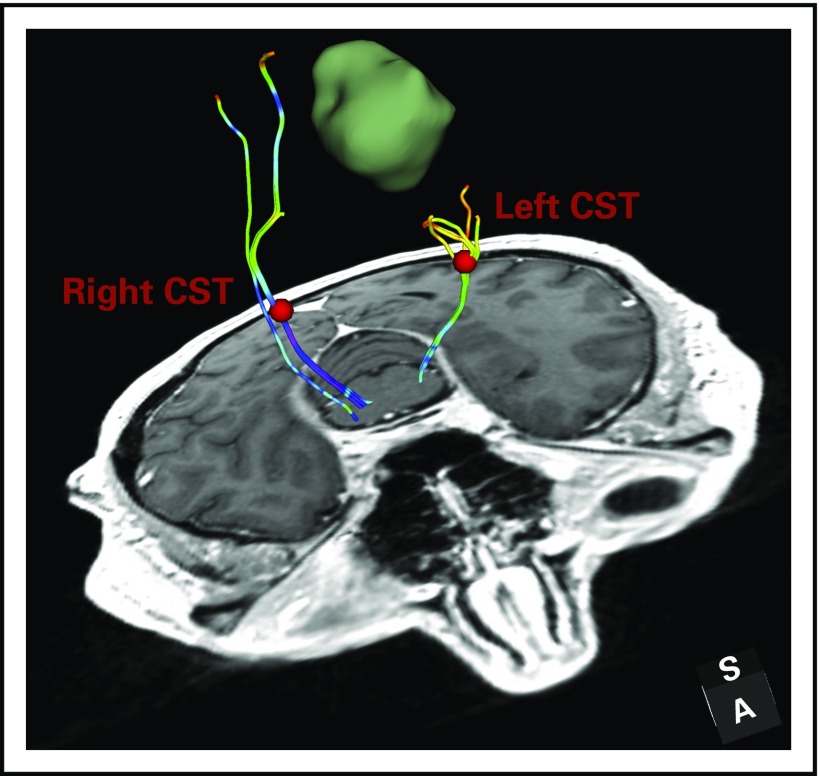
Diffusion tensor imaging (DTI) tractography analysis for mapping white matter connections in patient data set 1. DTI fiber tracking is seeded from two regions of interest that are placed in the corticospinal tract (CST) bundles in each hemisphere. The fiber tracts are colored by the fractional anisotropy (FA) values of the fiber points along the fiber tract. The mean FA of the fiber tract is 0.48 in the healthy hemisphere and 0.39 in the tumor-bearing hemisphere. The decreased FA is likely related to the tumor-related edema and/or infiltration.

DTI tractography can identify patient-specific fiber tracts for visualization. This can inform surgical planning to minimize morbidity associated with disruption of critical white matter connections. Figure 2 illustrates a DTI tractography analysis in patient data set 1, and the following paragraphs describe the workflow using SlicerDMRI.

#### Tractography fiber tracking and visualization.

SlicerDMRI supports single-fiber DTI tractography that performs fiber tracking by following the principal direction of the estimated diffusion tensors.^5^ Tractography seeding using two clinically relevant approaches is provided: seeding from an interactively visualized 3D object that can be manipulated by the user and seeding from an ROI that is stored as a label map, or mask. These two seeding strategies are provided in the TractographySeeding and TractographyROISeeding modules. Tractography visualization is performed using the TractographyDisplay module, where multiple tract visualization modes are provided (eg, fiber color by diffusion scalar measure, visualization as lines or tubes).

#### Tract quantification.

SlicerDMRI provides multiple tools to enable quantification of tract diffusion and geometric measures. For example, the TractographyMeasurement module allows for computation of multiple statistics (mean, maximum, etc) of diffusion-derived values of the fiber points along a tract. The TractographyToMask and LabelStatistics modules together enable the measurement of tract volume, and the WhiteMatterAnalysis package enables computation of the fiber length distribution of a tract.

## ADVANCED dMRI ANALYSES FOR NEUROSURGICAL PLANNING

Advanced analyses in SlicerDMRI include more sophisticated mathematical modeling of the white matter tissue, which enables depiction of crossing fiber tracts, and automated identification of critical fiber tracts, which enables rapid, standardized assessment of patient-specific neuroanatomy.

### Multifiber Tractography With Advanced Tissue Modeling

Conventional DTI tractography is often unable to fully trace fiber tracts in the peritumoral region^29^ because of the effects of edema and infiltration. DTI tractography is also limited to model only one fiber tract per voxel, despite that there are tens to hundreds of thousands of axons packed per square millimeter.^19,30^ DTI tractography cannot reconstruct fiber tracts that cross one another,^31-33^ such as the CST and AF. Modern multifiber tractography approaches can map a more-complex fiber architecture (eg, crossing fibers) to better trace important anatomic fiber tracts.^34^

SlicerDMRI supports advanced multifiber unscented Kalman filter (UKF) tractography,^35^ which provides an expanded range of multifiber models, including multitensor and multicompartment models. UKF tractography is more sensitive than standard single-tensor tractography, particularly in the presence of crossing fibers and peritumoral edema.^29,36-38^ Thus, UKF can better match the established understanding of surgical neuroanatomy than traditional DTI tractography. However, it should be noted that such comparisons are difficult because of the lack of ground truth, and tractography validation and standardization remain an open problem.^39,40^

**FIG 3.**
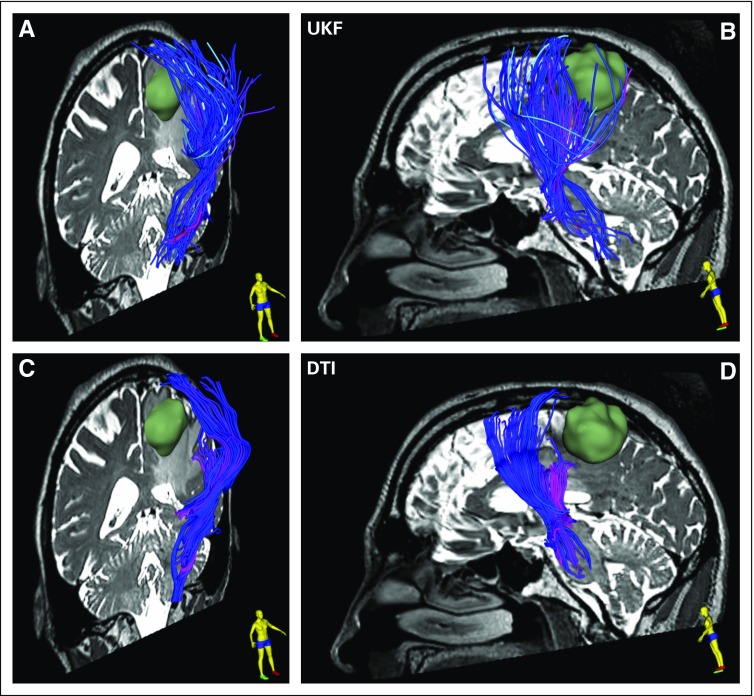
Single-tensor diffusion tensor imaging (DTI) *v* two-tensor unscented Kalman filter (UKF) tractography with a free water model of edema in patient data set 1. The tracts are visualized using tubes in three dimensions, with a diameter of 1 mm and colored by mean fiber orientation. (A and C) An oblique view from the right, featuring a coronal image, tumor in green, and corticospinal tract (CST) anterior to the tumor. The lateral projections of the CST that cross through edema and intersect with long anterior-posterior fibers are difficult to trace with conventional single-tensor DTI but can be traced using two-tensor UKF tractography. (B and D) A sagittal view of the relationship of CST and tumor on the basis of the two tractography methods. The UKF method with the free water model traces a seemingly larger volume of fibers within the edema that surrounds the tumor.

Figure 3 illustrates the advanced tractography analysis in patient data set 1, which features a comparison between single-fiber DTI tractography and the multifiber plus free 'water UKF method. The following paragraph describes the UKF tractography fiber tracking workflow using SlicerDMRI.

#### Multifiber UKF tractography with advanced tissue modeling options.

SlicerDMRI provides the InteractiveUKF and UKFTractography modules for fiber seeding from an interactively modifiable 3D object and an ROI mask, respectively. Advanced tissue modeling options, such as free water modeling^41^ and neurite orientation dispersion and density imaging,^42^ are available during UKF tractography fiber tracking.

### Automated Identification of Critical Fiber Tracts

**FIG 4.**
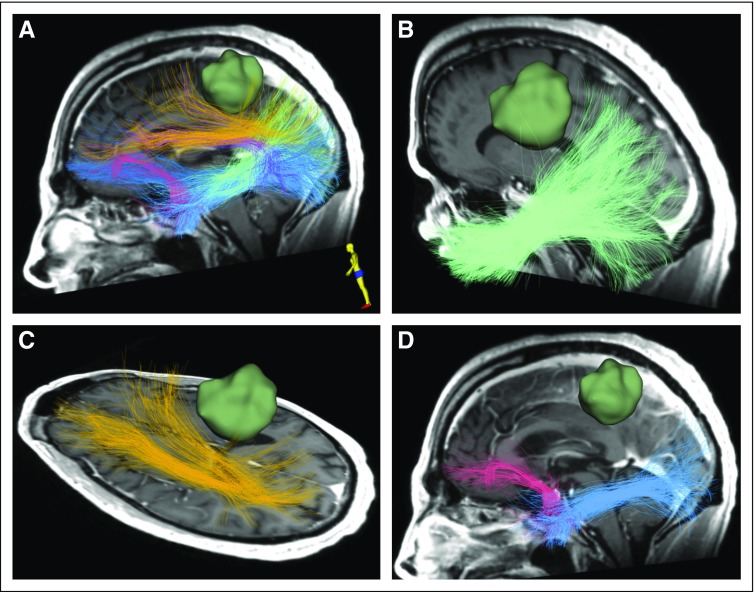
Automated identification of critical fiber tracts, including major language white matter connections, in patient data set 1. Two-tensor unscented Kalman filter method with the free water model is used for computing the whole-brain tractography data. Six language fiber tracts are identified and displayed, including the (A) arcuate fasciculus (purple), (B) inferior occipitofrontal fasciculus (dark blue), (C) middle longitudinal fasciculus (light green) and superior longitudinal fasciculus (yellow), and (D) uncinate fasciculus (pink) and inferior longitudinal fasciculus (light blue).

Conventional identification of critical fiber tracts requires expert manual interpretation, which is time consuming and has variable results across experts.^43^ Using a combination of machine learning and expert anatomic annotation, we created an anatomically curated white matter atlas.^44^ The atlas contains 58 deep white matter tracts, including major long-range association and projection tracts, commissural tracts, and tracts related to the brainstem and cerebellar connections. This atlas is released as an open source as part of SlicerDMRI.^45^ Using this atlas and our accompanying open-source software, we have demonstrated automatic identification of fiber tracts in patients with brain tumors, despite the challenges of edema and tract displacement as a result of mass effect.^44,46^ Figure 4 illustrates the application of this technology to automatically identify critical fiber tracts in patient data set 2, and the following paragraphs describe the workflow using SlicerDMRI.

#### Fiber clustering tract identification.

SlicerDMRI provides the WhiteMatterAnalysis Python package to perform automated tract identification using a data-driven fiber clustering pipeline. A one-step batch script is provided to run the pipeline for identification of fiber tracts according to the anatomically curated white matter atlas.^44,45, 47,48^ The 3D Slicer Ruler function can be used to measure the distance of the tracts to other image data (eg, a tumor 3D model) displayed in the 3D Viewer.

#### Recent advances using deep learning tract parcellation.

In addition to WhiteMatterAnalysis, in the near future, SlicerDMRI will also support fast tractography segmentation using deep learning techniques. This will be provided in the DeepWMA package. While achieving visually similar tract identification results to WhiteMatterAnalysis, DeepWMA performs much faster by leveraging deep learning and graphics processing unit computation (8 minutes *v* 1.5 hours on a data set containing 1 million fibers).^49^ This fast computation can motivate the use of automated analyses for intraoperative mapping of white matter connections.

## MULTIMODAL ANALYSIS FOR NEUROSURGICAL PLANNING AND GUIDANCE

There are multiple modalities available to identify both pathologic and eloquent tissue.^50^ Tractography is often combined with other modalities; thus, we discuss two of these adaptations to show their utility in brain tumor surgery.

### Multimodal dMRI and fMRI

Preserving function during brain tumor operations is associated with increased quality of life and overall survival.^51^ One broadly used neurosurgical planning tool is fMRI, which can aid in surgical planning and understanding where the boundaries of tumor and functional tissue exist.^52,53^ Although electrocortical estimation is the current gold standard in surgical brain mapping, approximately 25% of patients are not good candidates or fail awake surgery.^54^ When fMRI is combined with DTI, there is a combined sensitivity of techniques,^55^ which can even guide treatment with less invasive options, such as laser interstitial thermal therapy or radiation.^56^ A newer nontask-based technique, resting state fMRI (rsfMRI), is already being used for surgical planning^57^ and may be ideal for children or patients who are unable to perform a task. A prospective multi-institutional study to analyze fMRI, rsfMRI, and multifiber DTI techniques is needed.

**FIG 5.**
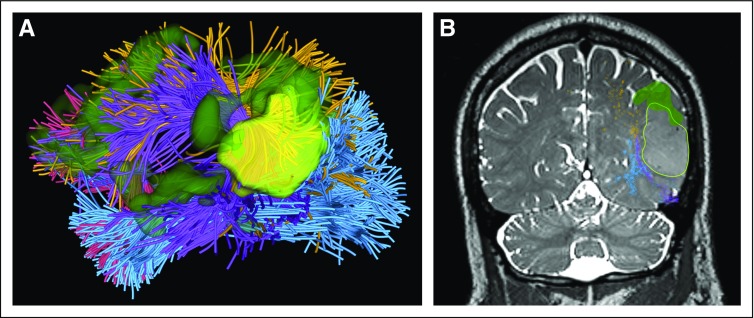
Visualization of multimodal diffusion magnetic resonance imaging (MRI) and functional MRI (fMRI) data in patient data set 2. (A) Three-dimensional (3D) visualization of tractography using two-tensor unscented Kalman filter method with free water modeling (tracts and colors as in Fig 4) with fMRI (dark green) and tumor (bright yellow green). (B) Intersection of the 3D visualization with a preoperative coronal T2-weighted MRI showing overlay of tracts, fMRI activations, and tumor surface. Recognition of the fMRI speech-related activations and the deeper subcortical arcuate fasciculus is particularly important in tumors that are adjacent to eloquent brain. Intraoperative visualization of these areas can aid the surgeon in the preservation of language and obtaining gross total resection.

Figure 5 illustrates the integration of multimodal dMRI and language-task fMRI data in patient data set 2 and features a visualization for presurgical brain structure and function mapping. The following paragraph describes this multimodel analysis using multiple software tools.

#### Multimodal dMRI and fMRI integrated visualization.

Anatomic fiber tracts identified using the SlicerDMRI WhiteMatterAnalysis tool are displayed together with functional activations computed from the fMRI data (see Selected Example Neurosurgical Patient Data Sets). Visualization of the multimodal data can be performed in either 2D mode (to show the intersection of 3D objects such as tracts and activations on a 2D image) or 3D mode through the 3D graphical user interface (Fig 5).

### Multimodal dMRI and Intraoperative Ultrasound

Another consideration during intraoperative identification of function is brain shift, which is the deformation of the brain that occurs during craniotomy and resection. As brain shift worsens during an operation, preoperative imaging becomes less reliable.^58^ The only commercially available option for intraoperative reregistration of a brain is intraoperative MRI, which is expensive, requires nonferromagnetic instruments, and adds up to 1 hour of operative time per scan. Because ultrasound resolution has improved, intraoperative ultrasound is increasingly used for updated real-time imaging.^59^ This allows the surgeon to better estimate the effect of brain deformation while also obtaining a real-time navigated view. Ongoing work is addressing quantification of this brain deformation such that the preprocedure magnetic resonance data, including tractography, can be updated to match the current brain configuration, thus improving the use of tractographic information to augment intraoperative surgical decision making.^60,61^

**FIG 6.**
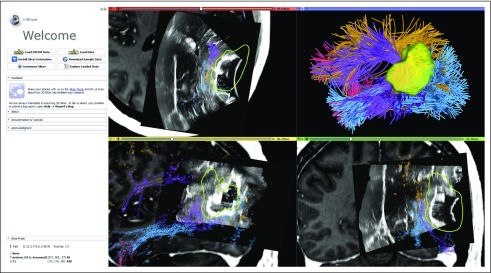
The 3D Slicer user interface that shows visualization of navigated intraoperative ultrasound (iUS) overlaid on preoperative T1-weighted magnetic resonance imaging with contrast along with the automatically identified language fiber tracts in patient data set 2 (tracts and colors as in Fig 4). The iUS image is used retrospectively here for a demonstration of software functionality. iUS information demonstrates brain shift that occurs after dural opening and likely displaces the arcuate fasciculus (purple), which can be seen at the edge of various parts of the resection cavity in each panel (top left, axial; bottom left, sagittal; bottom right, coronal). By integrating this brain shift information with tractography, the surgeon can make an updated estimate of how far the tracts might be. DICOM, Digital Imaging and Communications in Medicine.

Figure 6 illustrates the integration of multimodal dMRI and intraoperative ultrasound data in patient data set 2 and features a visualization for potential intraoperative real-time navigation. The following paragraph describes the workflow of this analysis.

#### Multimodal dMRI and ultrasound integrated visualization.

Anatomic tracts from presurgical dMRI data are computed using the SlicerDMRI WhiteMatterAnalysis tool and are registered to the presurgical anatomic T1-weighted image. Intraoperative ultrasound data are overlaid on the presurgical MRI data for an integrated visualization.

## HOW TO GET SlicerDMRI

SlicerDMRI is available for easy automated installation through the 3D Slicer Extension Manager, an app store for distribution of software extensions to 3D Slicer, with installation instructions and tutorials provided.^62^ SlicerDMRI is open source, so all code is available.^63^ User and software development support for SlicerDMRI is available through the 3D Slicer forums.^64^ SlicerDMRI has been used successfully for research with dozens of dMRI protocols from various vendors, with appropriate approvals from institutional review boards for each intended research use.

In conclusion, SlicerDMRI provides open-source and clinician-accessible software tools for dMRI research. In this work, we demonstrate SlicerDMRI functionality as an informatics tool to enable end-to-end dMRI analysis. We provide examples that demonstrate the use of SlicerDMRI to retrospectively analyze data from brain tumor resection.

## Data Availability

Patient data set 1 is publicly available upon request to fzhang@bwh.harvard.edu, whereas patient data set 2 will become publicly available online at the Cancer Imaging Archive (www.cancerimagingarchive.net) as part of the data release from the National Institutes of Health–funded project R01EB027134.
